# Hemozoin Inhibition and Control of Clinical Malaria

**DOI:** 10.1155/2014/984150

**Published:** 2014-02-09

**Authors:** Chibueze Peter Ihekwereme, Charles Okechukwu Esimone, Edward Chieke Nwanegbo

**Affiliations:** ^1^Department of Pharmacology and Toxicology, Faculty of Pharmaceutical Sciences, Nnamdi Azikiwe University, Awka 420281, Nigeria; ^2^Department of Pharmaceutical Microbiology and Biotechnology, Faculty of Pharmaceutical Sciences, Nnamdi Azikiwe University, Awka 420281, Nigeria

## Abstract

Malaria has a negative impact on health and social and economic life of residents of endemic countries. The ultimate goals of designing new treatment for malaria are to prevent clinical infection, reduce morbidity, and decrease mortality. There are great advances in the understanding of the parasite-host interaction through studies by various scientists. In some of these studies, attempts were made to evaluate the roles of malaria pigment or toxins in the pathogenesis of malaria. Hemozoin is a key metabolite associated with severe malaria anemia (SMA), immunosuppression, and cytokine dysfunction. Targeting of this pigment may be necessary in the design of new therapeutic products against malaria. In this review, the roles of hemozoin in the morbidity and mortality of malaria are highlighted as an essential target in the quest for effective control of clinical malaria.

## 1. Introduction

Malaria has plagued humankind since ancient times and is still a significant threat to half of the world's population [1]. Malaria is the fifth most common cause of death from infectious diseases worldwide (after respiratory infections, HIV/AIDS, diarrheal diseases, and tuberculosis) and the second in Africa, after HIV/AIDS [2]. Recent estimates show that as many as 3.3 billion people live in areas at risk of malaria in 109 countries or territories [3]. In addition to its health toll, malaria puts a heavy economic burden on endemic countries and contributes to the cycle of poverty people face in many countries. For example, it is estimated to have in Africa alone contemporaneous costs of at least US$12 billion per year in direct losses (e.g., illness, treatment, and premature death), but many times more than that in lost economic growth [4]. Malaria continues to be a major global health concern, with an estimated 243 million cases of malaria worldwide [5]. The vast majority of cases (85%) were in the African region, followed by Southeast Asia (10%) and Eastern Mediterranean regions (4%) [5]. Malaria accounted for an estimated 863 000 deaths in 2008 [5]. Malaria is a leading cause of child mortality in Africa, claiming a life nearly every 30 seconds [6]. Children are at highest risk for severe malarial illness and death during the first five years of life while their immune systems are developing [5]. Malaria causes anemia in pregnancy and is associated with a higher HIV-1 viral load in pregnant women [7, 8]. In Sub-Saharan Africa,* Plasmodium falciparum *is responsible for most cases of malaria.

The parasite's life cycle includes a cycle of asexual division in the human liver, another cycle of pigment-producing asexual division in red blood cells (RBCs), and a sporogenic development in the female anopheles mosquito. During the erythrocytic stage of development, the malaria parasite develops into a trophozoite form in a vacuole formed by the internal membrane of the host red cells. The trophozoite feeds on hemoglobin (Hb) by ingesting small amounts of red cell cytoplasm. The globin component is further digested into amino acids for the parasite's metabolic needs. However, heme is toxic to the parasite and is thus aggregated into the insoluble dark-brown crystal called hemozoin (Hz) which can accumulate in the parasitized red blood cell (pRBC). Both circulating and resident phagocytes acquire Hz through phagocytosis of pRBCs or free Hz crystals released after schizont rupture [9]. The presence of Hz disturbs normal cellular function and physiology of host whereas the parasite is less affected by it. The mechanism of inhibition of cellular function by Hz is thought to be the production of lipoperoxides from arachidonic acid [10, 11]. These compounds have been identified from Hz-fed macrophages and shown to inhibit macrophage function *in vitro* [12]. Furthermore, several studies have demonstrated that acquisition of malarial pigment by circulating monocytes and neutrophils is significantly associated with disease severity [13–16]. The Hz-monocyte complex has been associated with severe malaria anemia (SMA) [17], immunosuppression [9, 16], and cytokine dysregulation [18–20]. These findings demonstrate the pathological roles of Hz during malaria parasitaemia. However, recently researchers demonstrated that Hz may be an effective adjuvant for malaria vaccine [21–25]. This is because Hz was found to be a good ligand for Toll-like receptor 9 (TLR9) and induced both humoral and cellular immune responses in animals immunized with crude extract of Hz [21, 22].

Taken together, these findings suggest that Hz may be involved in both induction of immunity against malaria and the pathogenesis of the disease. In this brief review, we will evaluate reported roles of this pigment in the pathogenesis of malaria and provide the rationale for its targeting in antimalaria therapeutics development.

## 2. Hemozoin and Anemia

Structural abnormalities and extensive deposition of Hz were observed in the livers and bone marrows of children with SMA [26–28]. The abnormalities in these tissues indicate the pathological role of Hz in the development of SMA. Although there are many unexplained complex factors involved in the development of anemia during malaria infection, three mechanisms appear to be responsible for the problem, namely, direct destruction of infected red blood cells, increased destruction of unparasitized blood cells, and marrow suppression. Hz plays crucial roles in these three mechanisms.

### 2.1. Removal of Parasitized RBC

Anemia from *P. falciparum* malaria infection is usually responsible for severe morbidity and mortality in children and pregnant women in Sub-Saharan Africa. *P. falciparum* invades red cells of all ages [29] and digests Hb using parasite proteases [30] into small fragments consisting of about 20 different amino acids and free ferrous protoporphyrin IX, which is rapidly oxidized to heme [31]. Free toxic heme is rapidly oxidized to hematin and sequestered into inert, nontoxic crystalline Hz, which is present in the pRBC [32]. The presence of Hz in RBC results in appearance of antigenic molecules on membranes of these cells [33]. Recognition of Hz-containing RBC results in phagocytosis by circulating macrophages and removal from circulation. A study of the antigens exposed on the surface of pRBCs with different isolates of *P. falciparum* showed that the surface antigens could induce isolate-specific immunity [34]. Parasite-derived erythrocyte membrane protein (PfEMP1) facilitates rosetting and mediates the binding of pRBC to endothelial cells [35]. In another study which described clinically relevant cytoadhesive phenotypes of *P. vivax* isolates, it was observed that the intensity of rosetting was higher among anaemic individuals compared to non-anaemic and decreased with increasing haematocrit and haemoglobin levels [36]. Consequently, the study concluded that rosetting may contribute to development of anaemia. Previous studies involving *P. falciparum* suggest that rosetting and cytoadhesion contribute to anaemia [37, 38]. In a study in Kenya, the presence of Hz-containing monocytes in children infected with *P. falciparum* was shown to be associated with SMA [17].

### 2.2. Hemolysis of Unparasitized RBC

Trophozoites in the RBC grow and develop into schizonts which rupture the cell releasing merozoites and free Hz into blood circulation [39]. Circulating free Hz released after rupture of the RBC may be deposited on unparasitized RBC and cause lipid peroxidation of RBC membranes resulting in loss of deformability of the cell [40–42]. This change in membrane predisposes unparasitized RBC to increased sequestration and lysis in the spleen and other reticuloendothelial organs. A study investigating the mechanisms behind the structural and functional effects of haem products on infected and uninfected red cells showed that hemolysis induced by haematin was dose and time dependent [41]. Since red cells preincubated with haematin were more sensitive to haemolysis induced by hypotonic shock, low pH, H_2_O_2_, or haematin itself, the study concludes that the destabilising effect of haem products (haematin and beta-haematin) on red cell membrane may not result from oxidative damage of membrane lipids but from direct binding or incorporation to membrane. Furthermore, direct binding or incorporation of haem products to npRBC membrane is expected to initiate immunological responses and phagocytosis of npRBC.

Another mechanism involves 4-hydroxynonenal (4-HNE). Rosetting is a specialized form of cytoadherence of late hemozoin-containing pRBCs to npRBCs [43]. The aldehyde 4-HNE is synthesized in the parasite when iron, present in Hz, peroxidizes polyunsaturated fatty acids [42]. Uyoga et al. (2012) having studied the role of transfer of 4-HNE from parasitized to nonparasitized erythrocytes in rosettes concluded that the transfer plays a role in the phagocytic removal of large numbers of npRBCs and may be key to the SMA found in malaria patients [43].

### 2.3. Marrow Suppression

Hz has also been associated with direct inhibition of reticulocyte formation [15, 44]. Previous autopsy investigation in children that died from severe malaria demonstrated large deposition of Hz in bone marrow suggesting direct inhibition of erythropoiesis [28]. In addition, dyserythropoietic changes, including multinuclear erythroblasts, karyorrhexis, incomplete and unequal amitotic nuclear divisions, and cytoplasmic bridging, were also noted by Abdalla and colleagues in children with SMA in 1980 [45]. Similarly, proinflammatory cytokines such as TNF-*α* have been shown to inhibit all stages of erythropoiesis [46, 47]. Hz increases the secretion of TNF-*α* in both human and animal malaria infection [48–50].

In all, Hz directly promotes SMA through enhancing increased hemolysis of RBC and inhibition of erythropoiesis. It also indirectly promotes SMA by triggering production of inflammatory cytokines such as TNF-*α* and nitric oxide (see below).

## 3. Hemozoin, Cytokine, and Chemokine Dysfunction

Previous investigations had shown that ingestion of Hz by monocytes may enhance malarial pathogenesis by causing dysregulation in the production of cytokine, chemokines, and effector molecules, including TNF-*α*, interleukin IL-12, IL-10, macrophage inflammatory protein (MIP)-1*α*, MIP-1*β*, nitric oxide (NO), and prostaglandin (PG)-E_2_ [18–20, 33].

### 3.1. Tumor Necrosis Factor-Alpha

Systemic symptoms of malaria like fever occur after the rupture of malaria schizont [51, 52] and are caused by the release of proinflammatory cytokine TNF-*α* [35]. In both human and animal studies, Hz ingestion by mononuclear cells enhances production of TNF-*α* [48–50, 53] and nitric oxide [54, 55]. High systemic level of TNF-*α* is seen in acute *P. falciparum* malaria [56] and is believed to be protective by restricting parasitaemia [57]. However it also inhibits all stages of erythropoiesis and is associated with increased malaria pathogenesis [46, 58]. Similarly, inhibition of bone marrow and RBC destruction as a result of high TNF-*α* was reported in murine malaria [59, 60]. Higher TNF-*α* was also reported in fatal compared to nonfatal cerebral malaria in African children [61]. Similarly, higher TNF-*α* was seen in cerebral malaria compared to uncomplicated malaria [62]. In addition, elevated serum TNF-*α* level was associated with abortions [58, 63, 64]. Taken together, transient elevation of TNF may be beneficial since it enhances parasite clearance while sustained release of TNF in severe malaria is associated with increased malaria morbidity and perhaps mortality.

### 3.2. Nitric Oxide

Hz and pro-inflammatory cytokines including TNF-*α* increases the expression of nitric oxide synthase 2 (NOS_2_) gene and generation of nitric oxide (NO). Initial high levels of NO appear to be protective against severe malaria [65, 66]. However sustained high levels are associated with *P. falciparum* malaria anemia in children [55] by inhibiting erythropoiesis [67, 68].

### 3.3. Prostaglandins

In children with acute malaria, plasma prostaglandin E_2_ and COX-2 gene expression by PBMC are significantly reduced [69]. This is partly due to Hz ingested by PBMCs [70]. PGE_2_ inhibits TNF-*α* [71] and appears to decrease malaria severity. It also enhances erythropoiesis by inducing burst forming unit erythroid formation [72]. Inhibitors of PGE_2_ synthesis such as Hz, acetaminophen, and salicylates are associated with high levels of TNF-*α* and increased malaria severity and mortality [73–75]. In addition low circulating bicyclo-PG_2_/TNF-*α* is associated with decreased Hb concentration [55]. Hz has also been known to affect serum levels of IL-10 [33, 76] and IL-12 [77]. IL-10 may be increased in severe malaria while low IL-12 was also reported to be associated with increased malaria disease severity in children [13, 78].

In summary, Hz appears to trigger pathological levels of pro-inflammatory cytokines and chemokines like TNF-*α*, NO and at the same time reducing the level of more beneficial IL-12 and prostaglandin E_2_. The role of anti-inflammatory cytokine IL-10 in malaria appears to enhance parasitemia since the Th1 immunological responses against the parasite are inhibited and IL-12 secretion is also suppressed by this cytokine. Taken together, Hz plays important role in deregulation of pro- and anti-inflammatory cytokines during malaria resulting in altered immunological responses to the disease, anemia, and host tissue damage.

## 4. Immunosuppression

Suppression of innate immune response during malaria infection has been reported in previous studies [9, 79]. One of the reported mechanisms is the suppression of dendritic cell (DC) function [33]. Dendritic cells play important roles in innate and adaptive immune responses. In contact with pathogen, these cells phagocytize the antigen, undergo a process of maturation, upregulate the requisite molecules and present to NK cells and naive and memory T and B lymphocytes. Ingestion of pRBC by DCs has been reported to impair the natural ability of DCs to stimulate both allogenic and antigen-specific T-cell immunity [80, 81]. This inhibition of DCs function is probably partially due to phagocytized Hz in pRBC. Moreover, Hz direct inhibition of DC maturation [33, 82] and suppression of general leucocytes proliferative responses [76] were also reported in previous studies. Similarly, ingestion of Hz by monocytes, macrophages, and neutrophils had been known to affect the functions of these cells resulting in defective phagocytosis and expression of MHC Class II, CD54, and CD11c [9, 79]. Specifically, decrease in IL-12 during malaria may be partly responsible for decreased immunity to the parasite. As mentioned above, low IL-12 has been associated with severe malaria infection in children [13, 78]. The derangement in the innate immune responses ultimately results in defective adaptive immune responses. As mentioned earlier, suppression of DC function is associated with failure of adequate priming and presentation of malaria antigens to CD4+ and CD8+ cells for appropriate Th1 and Th2 immune responses.

The suppression of immunity during malaria infection may be responsible for associated clinical problems. For instance, studies have demonstrated increased viral load in HIV patients [83] as a result of suppression of CD8+ function. Otieno et al. demonstrated increased SMA in HIV-1 positive infants and children in Kenya [84]. Also ineffective CD8+ function during malaria was linked to the association of Burkitt's lymphoma and endemic malaria [85, 86]. In addition, frequent association of bacteremia in children with clinical malaria was reported previously [87–89]. In all, presence of circulating malaria pigment in macrophages and neutrophils was associated with poor prognosis [90] or increased morbidity [91, 92].

On account of the global inhibition of innate and adaptive immune responses, Hz was described in previous studies as a potent immunosuppressant [93, 94]. However, these findings have been challenged by recent reports that demonstrated the potent immunogenicity of crude extract of Hz. These studies reported that Hz is an effective TLR9 ligand and can enhance immunity against malaria [50, 95].

## 5. Other Harmful Effects of Hemozoin

A number of harmful effects have been documented or associated with Hz. Hz compromises the functions of human monocytes, as previously noted, and this dysfunction has been related to its lipoperoxidation products, namely, 15-hydroxyeicosatetraenoic acid (15-HETE) and 4-HNE [96]. Hz has also been associated with malaria-associated acute respiratory distress syndrome (MA-ARDS). By quantifying Hz in the lungs and measuring the disease parameters of MA-ARDS, a highly significant correlation between pulmonary Hz concentrations, lung weights, and alveolar edema was demonstrated [97]. Another study has shown that Hz is implicated in cerebral malaria since it modulates matrix metalloproteinases and induces morphological changes in human microvascular endothelium [98]. Histological examination in the study revealed that human microvascular endothelial cell line (HMEC-1) treated with natural Hz appeared elongated instead of polygonal and formed microtubule-like vessels on synthetic basement membrane.

## 6. Summary

One of the factors responsible for malaria pathogenesis is the suppression of host immune responses. This suppression enhances parasitemia and diminishes the host immune response to malaria-expressed proteins and other pathogens. Hz plays both direct and indirect roles in orchestrating this immunosuppression which may be important for the survival of the parasite and completion of its life cycle in the human host [99]. Blocking of Hz formation will ultimately increase immune mediated parasite clearance and can prevent formation and transmission of the gametocytes. The host can also maintain immunological surveillance to other pathogens. Development of new chemotherapeutic agents that will prevent the formation of the pigment in pRBC may greatly reduce morbidity associated with malaria. Association of Hz and SMA has been demonstrated in several previous studies (see above).

Use of agents that will prevent formation of the pigment will prevent Hz-induced inhibition of erythroid precursors and also reduce the production of other mediators of SMA. Chloroquine, quinine, and artemisinin block the formation of Hz from heme [21, 22, 95]. Perhaps, reduction of morbidity and mortality with these drugs may be attributable to decreased circulating levels of Hz. In developing countries where malaria is endemic, effective targeting of Hz may greatly reduce high morbidity and mortality presently associated with the disease. In this regard, development of new therapeutics that will block formation of Hz by the parasite will be necessary. Modification of quinine, chloroquine, or artemether to overcome parasite resistance may also achieve the same goal. Furthermore, it may be important to investigate the prevalence of anti-Hz immunity in healthy, mild, and severe malaria in people living in malaria endemic regions. Similarly, evaluation of crude extract of Hz as an adjuvant for human application requires further studies. This may provide the rationale for the inclusion of Hz as a component of candidate malaria vaccines. The applications of these potential Hz targeting measures warrant further studies.
